# Evaluation of the Antimicrobial Effect of Bioprotective Lactic Acid Bacteria Cultures Against *Listeria monocytogenes* in Vacuum-Packaged Cold-Smoked Rainbow Trout (*Oncorhynchus mykiss*) at Different Temperatures

**DOI:** 10.3390/foods14111951

**Published:** 2025-05-30

**Authors:** Javier Sánchez-Martín, Salud María Serrano-Heredia, Arícia Possas, Antonio Valero, Elena Carrasco

**Affiliations:** Departamento de Ciencia y Tecnología de los Alimentos, UIC Zoonosis y Enfermedades 10 Emergentes (ENZOEM), Campus de Excelencia Internacional en Agroalimentación (CeiA3), 11 Universidad de Córdoba, Campus Rabanales, Edificio Darwin-Anexo, E-14071 Córdoba, Spain; t52sehes@uco.es (S.M.S.-H.); g12mepoa@uco.es (A.P.); bt2vadia@uco.es (A.V.); bt2cajie@uco.es (E.C.)

**Keywords:** *Leuconostoc carnosum*, *Lactococcus lactis*, *Lactobacillus pentosus*, *Lactobacillus plantarum*, packaging, sensory analysis

## Abstract

The growing demand for Ready-to-Eat (RTE) fish products increases the need for effective safety measures against *Listeria monocytogenes*, a pathogen associated with high fatality rates. This study evaluated the bioprotective potential of lactic acid bacteria (LAB) strains, including probiotic ones, against *L. monocytogenes* in cold-smoked rainbow trout. Two LAB cocktails were tested: a commercial mix (LC–LL) and a vegetable-derived mix (LAB2–LP15). LC–LL effectively inhibited *L. monocytogenes* at both static (5 °C) and dynamic (4–20 °C) conditions by the inhibitory effect of the bacteriocin leucocin (≈4 log unit growth inhibition). In contrast, LAB2–LP15 was effective only at 5 °C (≈2 log unit growth inhibition), maintaining the best sensory characteristics. These findings support the use of LAB as natural bioprotective agents in RTE fish, combining food safety and sensory preservation.

## 1. Introduction

In recent years, there has been a remarkable surge in consumer demand for Ready-to-Eat (RTE) foods, reflecting notable shifts in dietary and lifestyle habits of increasingly health-conscious consumers. RTE fish and fishery products cater to a growing market of high-quality protein, omega-3 fatty acid-packed alternatives, aligning with the increasing awareness about the nutritional benefits associated with fish consumption [1]. In addition, the production of RTE fish from aquaculture is expected to rise, as estimates for the next few years foresee a significant increase in the consumption of fish from this sector compared to wild fishing [2].

RTE fish represents a food commodity with the potential to support the proliferation of harmful microorganisms such as *L. monocytogenes* [3]. *L. monocytogenes* is one of the most relevant foodborne pathogens since it is psychrotrophic, ubiquitous in the environment, and capable of forming biofilms [4], representing a source of contamination in food processing chains. In 2022, a total of 2738 reported cases of invasive human cases of listeriosis were confirmed, ranking it the fifth most commonly reported zoonosis in humans in the European Union (EU) and increasing its incidence by 15.9% compared to 2021, the highest rate since 2007 [5]. The EFSA report presented the highest numbers ever found with regard to the percentages of *L. monocytogenes* positives in RTE fish products (2.3%), at the manufacturing stage (2.6%), and in fishery products (2.5%) [5]. Annual reports compile several outbreaks in RTE fish and fishery products, with 17 instances documented in Europe between 2022 and 2023 and 10 fatal cases from 2019 to 2023 [6]; some of the occurrences have been reported in Andalusia (Spain) [7].

RTE fish and fishery products require low-temperature management and strict hygiene control against contamination [8], which, in some instances, is insufficient to efficiently eliminate the presence of *L. monocytogenes* in industrial environments [9,10]. In this sense, other factors leading to an improvement of food safety and an extension of shelf-life, have been historically implemented, such as the use of modified atmosphere, application of smoking treatments, or addition of sodium chloride (NaCl), organic acids, or brine-cured preparations [11].

In the search for strategies to carry out more efficient control of *L. monocytogenes*, bioprotection emerged as a promising technology for shelf-life extension, offering a viable solution to enhance food safety and quality while extending product freshness. A bioprotective culture could be defined as a culture of live microorganisms used to ensure the hygienic quality of foodstuffs [12] without adversely affecting sensory or technological properties [13]. Among them, LAB are the most extensively studied bacterial group. Bioprotective cultures and their metabolites have been widely assessed in different RTE fish and fishery products (especially salmon and surimi) against *L. monocytogenes*, with *Lactobacillus*, *Lactococcus*, *Carnobacterium*, or *Enterococcus* being the most commonly reported species with recognized antilisterial action due to the capacity for bacteriocin production, organic acid production and acidification, or nutrient competition [14,15,16,17]. However, most studies in the literature have not considered the probiotic potential of some LAB, such as protection against gut infections, antioxidant activity, prevention of diabetes type I and II, and improvement of cognitive processes, as demonstrated for *Lactobacillus* spp. and *Bifidobacterium* spp. [18,19]. For example, *Lb. pentosus* (LPG1; LAB119; LAB2) and *Lb. plantarum* (LP15) strains isolated from vegetable pickles not only exert a bioprotective effect but also have demonstrated probiotic activity [20], thereby increasing the added value of the product.

The aim of this study was to assess the inhibitory activity of LAB strains with probiotic potential on *L. monocytogenes* in cold-smoked rainbow trout fillets through a challenge test at static (5 °C) and dynamic (4–20 °C) temperature profiles and packaged with two types of film. The antilisterial activity was further evaluated in comparison with a commercial bioprotective culture at the same storage conditions.

## 2. Materials and Methods

### 2.1. Bacterial Strains

A total of four LAB strains were used in the study. From them, three strains belonged to the species *Lb. pentosus* (LAB2, LPG1, and LAB119), and one to the *Lb. plantarum* species (strain LP15). All strains, kindly provided by the Institute of Fats (Spanish National Research Council, CSIC), were isolated from vegetable pickles, having been tested for probiotic potential such as the anti-inflammatory effect properties, evidenced by their ability to significantly reduce the production of pro-inflammatory cytokine interleukin-8 (IL-8), or the modulation of the human immune system by stimulating the production of key immunomodulatory molecules (IL-6 and IL-10) [20]. LAB strains were reconstituted in de Man, Rogosa, and Sharpe Broth (MRS, Oxoid, UK) and subsequently stored in cryovials with 25% glycerol (*v*/*v*) at −80 °C.

LC-LL, a commercial mixture of two species, *Leuconostoc carnosum* and *Lactococcus lactis* subsp. *lactis*, was kindly provided by the Chr. Hansen company (Hoersholm, Denmark). LC-LL cultures were received in lyophilized frozen pouches, which were stored at −20 °C until use.

Regarding *L. monocytogenes*, two cocktails of strains were used. Firstly, to evaluate the antilisterial potential of the selected *Lb. pentosus* and *Lb. plantarum* strains, an in vitro assessment with the spot-on-the-lawn methodology was performed using a five-strain cocktail of *L. monocytogenes* CECT4032, CECT5366, CECT935, CECT5725, and CECT7467 from the Spanish Culture Type Collection (CECT, Valencia, Spain) (cocktail I). The origins of the strains are, respectively, as follows: three clinical isolates—one case of meningitis after eating soft cheese (UK), one case of human listeriosis (unknown country), and one case of spinal fluid of a child with meningitis (Germany)—and two food isolates—one in chicken (UK) and one in poultry (UK).

Secondly, an in vitro test in broth and a challenge test on cold-smoked rainbow trout fillets were carried out with a three-strain cocktail of *L. monocytogenes*: LMG23773 from the Belgian Coordinated Collections of Microorganisms (BCCM, Belgium) and isolated from smoked salmon; 12MOB102LM isolated from RTE salmon; and 12MOB107LM isolated from rainbow trout, acquired from French Agency for Food, Environmental and Occupational Health & Safety of France (ANSES) (cocktail II). Strains were selected based on their origin in RTE fish products. They were previously used in a study on RTE smoked salmon paté [21]. The bacteria were stored in cryovials in tryptone soya broth (TSB, Oxoid, UK) with 25% glycerol (*v*/*v*) at −80 °C until use.

### 2.2. In Vitro Assessments

#### 2.2.1. Spot-on-the-Lawn Experiments

In vitro assessment was performed using a spot-on-the-lawn assay. A culture of *Lb. pentosus* and *Lb. plantarum* strains was inoculated on MRS agar by placing three drops, each containing 2 µL, at the vertices of an equilateral triangle. The culture had a concentration of 8 log CFU/mL, previously adjusted to 0.5 turbidity of McFarland standard, and the plates were incubated at 30 °C with 10% CO_2_ for 16–18 h. Then, a soft tryptic soy agar (TSA) agar layer, previously inoculated with *L. monocytogenes* at 7 log CFU/mL, was rapidly poured onto the surface of the MRS plates with the three LAB spots. Plates were left to dry for 30 min and then incubated at 37 °C for 44–48 h. The inhibition diameter (mm) was measured with the help of a caliper (RS PRO Electronic Digital Calliper, London, UK).

#### 2.2.2. Experiments at Refrigeration Conditions

*Lb. pentosus* and *Lb. plantarum* strains were co-inoculated in TSB vials with the *L. monocytogenes* cocktail II at initial concentrations of 2–3 log CFU/mL for both microorganisms (LAB and pathogens). Vials were stored at two different refrigeration conditions, 5 °C and 8 °C. The interactions were monitored by culturing and counting LAB and *L. monocytogenes* on MRS agar at 30 °C with a 10% CO_2_ atmosphere and Oxford agar (Oxoid, UK) over 44–48 h, respectively. In addition, a control inoculated with *L. monocytogenes* cocktail II only was cultured on Oxford agar over 44–48 h. Samples were analyzed every 2–3 days over 21 days.

### 2.3. Challenge Test of LAB Against L. monocytogenes Cocktail in Cold-Smoked Farmed Rainbow Trout

The inhibitory activity of the selected LAB strains was assessed against the cocktail II of *L. monocytogenes*.

#### 2.3.1. Sample Preparation

Rainbow trout used in this study was produced by a fish farm located in Andalusia (Spain) and processed in a smoking production facility (Andalusian Aquaculture Technological Centre, CTAQUA) as described next. The product was submitted to a curing and smoking process using a solution of salt to sucrose (3:1) for 3 h. This was followed by 1 h of cold smoking at 30 °C with 80% relative humidity. Then, after filleting (80–100 g per fillet), they were vacuum-packaged in commercial polyamide/polyethylene (PA/PE) plastic bags and refrigerated before being sent to the laboratory. At the start of the experiments, the fillets were cut into small portions of approximately 10 g and arranged in trays for inoculation.

#### 2.3.2. Experimental Design

Batches of vacuum-packaged smoked rainbow trout fillets were prepared as follows: batch 1: *L. monocytogenes* cocktail II + LC-LL; batch 2: *L. monocytogenes* cocktail II + LPG1-LAB119 cocktail; batch 3: *L. monocytogenes* cocktail II + LAB2-LP15 cocktail; batch 4: *L. monocytogenes* cocktail II only; batch 5: LC-LL only; batch 6: LPG1-LAB119 cocktail only; batch 7: LAB2-LP15 cocktail only; and batch 8: control samples without inoculation.

Two storage conditions were tested in this study: one static condition at 5 °C for 22 days and a dynamic temperature profile simulating temperature abuse, i.e., starting at 4 °C for 7 days, increasing to 8 °C for the next 7 days, a 2-h abuse of 20 °C, and finally another 8 days at 8 °C. Samples were vacuum packaged for both storage conditions.

The packaging effect was studied on batch 1 (with the highest inhibition effect observed against *L. monocytogenes*) and batch 4. For this, two different packaging films were tested: the first packaging film was the regular commercial bags made of PA/PE (oxygen permeability lower than 30 cm^3^/m^2^ in 24 h), called permeable (P), while the other had a different polymer EVOH/PA/PE composition to reduce the permeability in a range under 2.5 cm^3^/m^2^ in 24 h, called non-permeable (NP).

Growth of *L. monocytogenes* on rainbow trout was characterized and modeled by predictive microbiology biogrowth software [22] for batch 3 (*L. monocytogenes* cocktail II + LAB2-LP15) and batch 4 (*L. monocytogenes* cocktail II without LAB) at 5 °C for both batches and at dynamic temperature conditions for batch 4.

Experiments, batches, and analyses were carried out in duplicate.

#### 2.3.3. Inoculum Preparation

A volume of 100 µL was taken from the cryovials to inoculate sterile TSB tubes with *L. monocytogenes* strains and sterile MRS tubes with LAB strains. Then, cultures were incubated 20–24 h at 37 °C and 20–24 h at 30 °C with a 10% CO_2_ atmosphere, respectively. Two consecutive 20–24 h subcultures were performed under the same time and temperature conditions to achieve the optimal physiological state of the microorganisms. Subsequently, *L. monocytogenes* strains were adapted to cold conditions by growing them at 10 °C for 72 h before inoculation. Cocktail II of *L. monocytogenes* was prepared by mixing 1 mL of each strain culture, setting the optical density to 1 reading at 650 nm, and diluting with saline solution 0.85 (*v*/*v*) (1:1000) to reach the targeted inoculum concentration (6 log CFU/mL). LAB cocktails were prepared by inoculating flasks with 100 mL of MRS broth, and then, the flasks were incubated in a 10% CO_2_ atmosphere at 30 °C for 20–24 h. LAB cultures were centrifuged twice at 3,606 RCF for 5 min and resuspended in saline solution (0.85% *w*/*v*) before spray suspension preparation. For *Lb. pentosus* and *Lb. plantarum* cocktails (LPG1-LAB119; LAB2-LP15), 50 mL of the above suspension cocktails were mixed with 350 mL of glucose solution 1% (*w*/*v*) in a sterile spray flask (7.5 log CFU/mL final concentration). The LC-LL culture was reconstituted from a lyophilized pouch following the manufacturer’s instructions, diluting 200 g of pouch content in 3 L of sterile distilled water. From this, 50 mL was mixed with 350 mL of sterile distilled water in a sterile spray flask (7.5 log CFU/mL final concentration).

#### 2.3.4. Rainbow Trout Fillets Inoculation

LAB inoculation (10 µL) was performed by spraying both sides of fillet portions six times each side (to set a concentration of 6 log CFU/g in the product). Once sprayed, the trout pieces were dried for 15 min inside a laminar flow cabinet. After this, 10 µL of the suspension of *L. monocytogenes* cocktail was inoculated on the surface of the trout portions. Samples were dried again for 15 min and vacuum packaged in commercial plastic bags before storage at static (5 °C) and dynamic (4–20 °C) temperatures.

#### 2.3.5. Microbiological, Physicochemical and Sensory Analysis

The microbiological analyses were conducted over 22 days, with testing every 2 days during the initial 14 days and every 3 days afterwards. At each analysis time, vacuum-packaged samples were placed into sterile blender bags with filters and ten-fold diluted with sterile peptone water solution 1% (*w*/*v*). Samples were homogenized in a blender (BagMixer Interscience, London, UK) for 90 s. Next, samples were serially diluted (in sterile saline solution 0.85% (*w*/*v*)) and surface cultured on Oxford agar for *L. monocytogenes* at 37 °C, 48 h, and on MRS agar for LAB enumeration at 30 °C, 48 h with 10% CO_2_ atmosphere.

As physicochemical parameters, pH (Hanna pH meter HI2020-48, Padova, Italy) and aw (Water Activity Meter AQUALAB 4TE, Madrid, Spain) were measured in trout samples from batch 8 (without inoculum) and from batches 5, 6, and 7, inoculated with only LAB cocktails (LC-LL, LPG1-LAB119, and LAB2-LP15, respectively), to assess possible significant variations depending on the strains.

Sensory analysis was designed following the Quality Index Method (QIM) [23] with some modifications to evaluate the impact of LAB cultures on the organoleptic characteristics of cold-smoked rainbow trout stored at 5 °C. To carry out the analysis, 10 g-fish samples of batches 5, 6, 7, and 8 were used. The sensory parameters evaluated included color (assessed visually), odor (by olfactory inspection), taste and salty flavor (by sampling), texture (by applying pressure to the fillet), and lipid oxidation (evaluated visually). Each parameter was rated on a scale from 1 (poor quality) to 5 (excellent quality), with scores of 4–5 indicating ‘very good’ quality, 3.0–3.9 ‘good’ quality, and 1.0–2.9 ‘unacceptable’ quality. The sensory assessment was performed on days 0, 5, 10, 14, 19, and 24 of storage. At each time, a single portion of fish from each batch was individually presented on regular Petri dishes and identified with three random numbers served under white light. The sensory panel was composed of a group of 12 semi-trained panelists from the Department of Food Science and Technology of the University of Córdoba (Spain).

### 2.4. Growth Modelling

Growth of *L. monocytogenes* on rainbow trout was characterized and modelized by biogrowth software (https://foodmicrowur.shinyapps.io/biogrowth/ (accessed on 27 May 2025)) for batch 3 (*L. monocytogenes* cocktail II + LAB2-LP15) and batch 4 (*L. monocytogenes* cocktail II) at 5 °C for both batches and at dynamic temperature conditions for batch 4. Estimated parameters were λ (lag time; h), µ_max_ (maximum growth rate; 1/h), N_0_ (initial cell concentration at time 0; log CFU/g) and N_max_ (maximum population density; log CFU/g). The growth data acquired were modelized and represented using Baranyi & Roberts equations when significant growth of *L. monocytogenes* was observed. 2.5. Statistical Analysis

Kruskal–Wallis tests (*p* < 0.05) were used to assess significant differences in the growth of *L. monocytogenes* cocktail II between different batches. Statistical differences in sensory evaluation for batches 5, 6, 7, and 8 were determined by the T2 Tamhane’s test (*p* < 0.05) for multiple comparative analysis.

## 3. Results

### 3.1. Screening of LAB Strains

**Figure 1:**
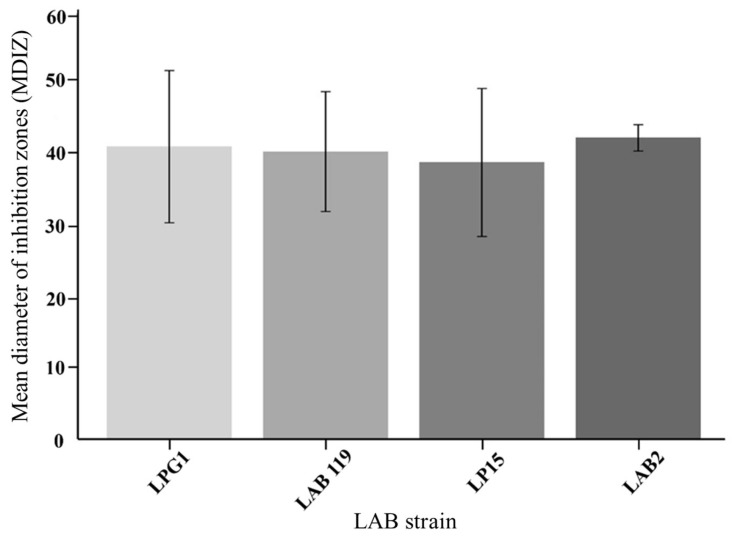
Mean diameter of inhibition zones (mm) of four LAB strains against *L. monocytogenes* five strains cocktail.

The mean diameter of inhibition zones (MDIZ) in the in vitro experiment is shown in Figure 1. The inhibition potential of the four LAB strains tested was favorable against the cocktail of *L. monocytogenes* strains, as demonstrated by the spot-on-the-lawn assay. Notably, the results revealed remarkable inhibition zones of approximately 40 mm. These results align with previous studies using LAB in fishery products with lower inoculum concentrations [24,25].

**Figure 2:**
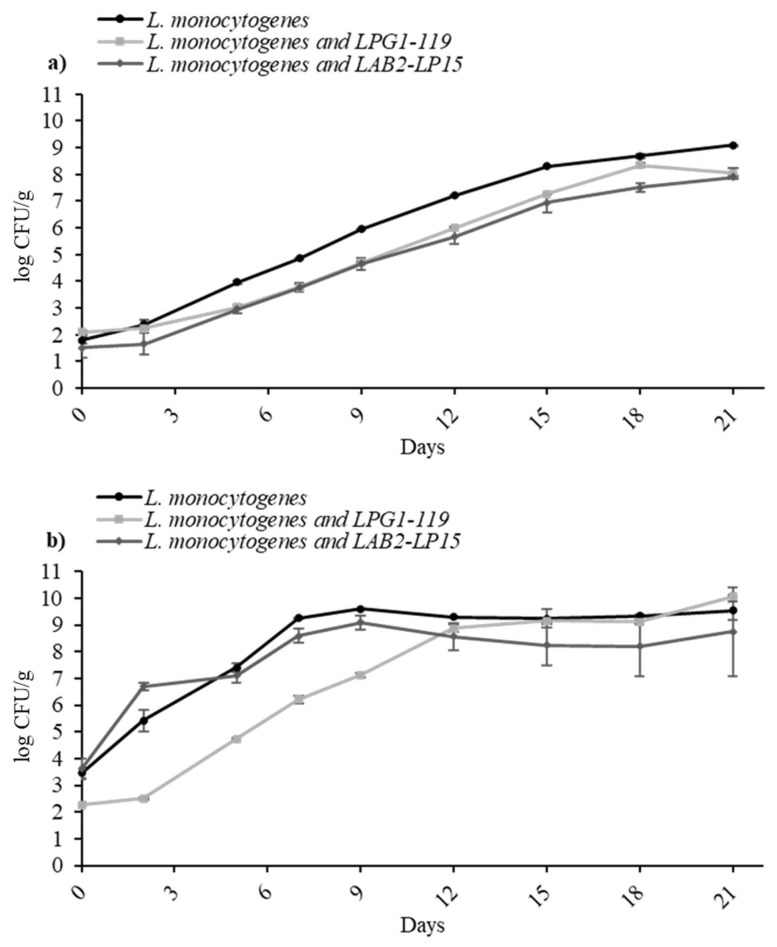
Growth of *L. monocytogenes* cocktail II in TSB at 5 °C (**a**) and 8 °C (**b**) in mono-culture and in co-culture against two LAB cocktails, LPG1-LAB119 and LAB2-LP15.

To know the effect of selected LAB, two studied LAB cocktails (LPG1-LAB119; LAB2-LP15) were used at refrigeration conditions (5 °C and 8 °C) against the cocktail II of *L. monocytogenes*. Figure 2 presents the growth curves of the *L. monocytogenes* cocktail in mono-culture (control) and in co-culture against LAB cocktails in TSB. At 5 °C, all LAB strains demonstrated a marginal inhibition of the pathogen, effectively diminishing the growth potential by approximately 1 log CFU/mL compared to the control samples. At 8 °C, LAB cocktail LAB2-LP15 seems to be slightly more effective than LPG1-LAB119, although with no significant differences (*p* > 0.05).

### 3.2. Inhibitory Effect of LAB Cultures

**Figure 3:**
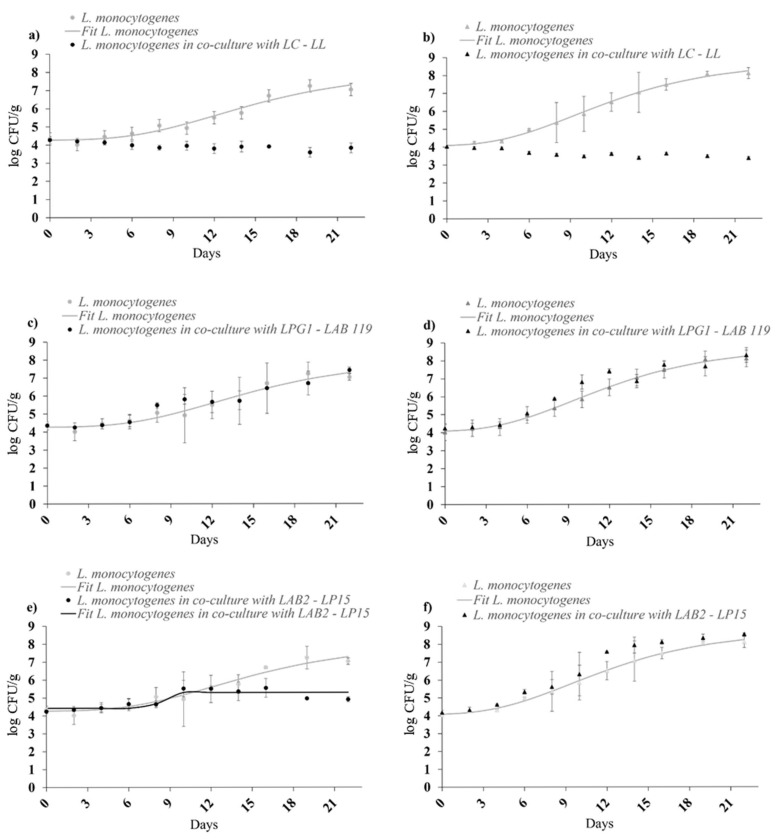
Growth/survival of *L. monocytogenes* cocktail II on cold-smoked rainbow trout at 5 °C in mono-culture and in co-culture against (**a**) LC-LL, (**c**) LPG1-LAB119, and (**e**) LAB2-LP15; and at dynamic profile temperature (4–20 °C) in mono-culture and in co-culture against (**b**) LC-LL, (**d**) LPG1-LAB119, and (**f**) LAB2-LP15.

The inhibitory effect of the LC-LL LAB cocktail was evident under static and dynamic temperature conditions (Figure 3a,b). When significant growth of *L. monocytogenes* was observed (batches 3 and 4), the growth model of Baranyi & Roberts [26] was adjusted to growth data. These LAB led to a slight reduction in the final concentration of *L. monocytogenes* compared to the initial level, with a reduction of 0.5 log units. In mono-culture, *L. monocytogenes* exhibited a notable growth potential. LPG1-LAB119 strains (Figure 3c,d) did not present a significant inhibitory effect (*p* > 0.05). LAB2-LP15 showed a positive effect at 5 °C (Figure 3e), maintaining a final concentration < 5 log CFU/g. At dynamic storage (Figure 3f), the bioprotective effect was not observed. No significant variations (*p* > 0.05) were observed in pH and water activity values throughout the study period.

### 3.3. Mono-Culture and Co-Culture Modelling

Table 1 and Table 2 show kinetic parameters. At 5 °C, the lag phase (λ) of *L. monocytogenes* was extended by 66.5 h when co-cultured with LPG1-LAB119. Final concentrations were reduced by 0.65 and 1.08 log CFU/mL with LPG1-LAB119 and LAB2-LP15, respectively. At 8 °C, only LAB2-LP15 showed significant reduction (around 1 log unit).

In trout, *L. monocytogenes* growth rate in mono-culture aligned with the literature. With LC-LL, levels decreased below the initial, and model fitting was not applicable. Only LAB2-LP15 showed significant differences in N_max_ at 5 °C (2 log unit reduction). Under dynamic conditions, *L. monocytogenes* had faster growth (higher rate and final density).

### 3.4. Sensory Evaluation

**Figure 4:**
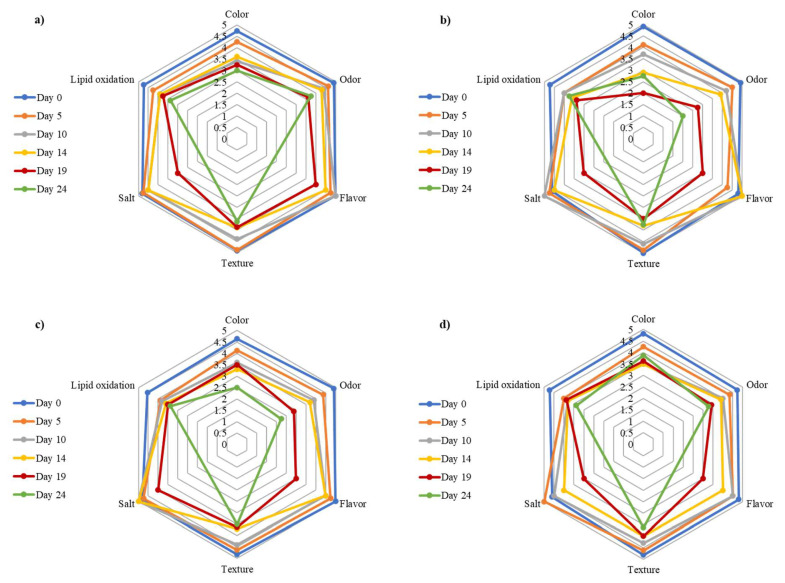
Representation of sensory attributes in vacuum-packaged smoked rainbow trout stored at 5 °C from batches (**a**) 5, (**b**) 6, (**c**) 7, and (**d**) 8. On day 24, flavor and salty taste were not evaluated for sanitary reasons.

Figure 4 shows scores from batches stored at 5 °C. Attributes declined over time as expected. Significant differences (*p* < 0.05) were observed in odor and color for LC-LL and LPG1-LAB119 (Table 3). These fell below three points. Control and LAB2-LP15 maintained parameters above 3, with the best results in color and odor. With regard to texture and oxidation attributes, there were no significant differences between batches (*p* > 0.05), although the value for LC-LL and LPG1-LAB119 fell below three points.

### 3.5. Effect of Packaging

**Figure 5:**
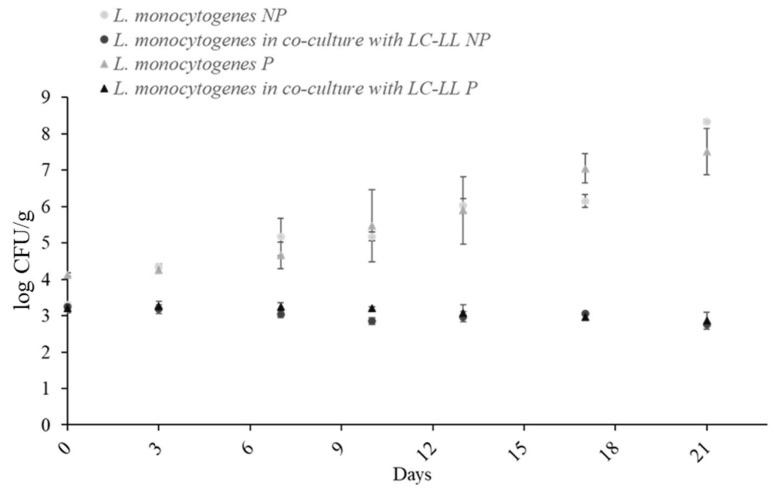
Growth of *L. monocytogenes* cocktail II on cold-smoked rainbow trout packed with two different films, NP and P, at dynamic temperature (4–20 °C) in mono-culture and in co-culture against LC-LL. NP: oxygen permeability < 2.5 cm^3^/m^2^ in 24 h; P: oxygen permeability < 30 cm^3^/m^2^ in 24 h.

Batch 1 (co-culture with LC-LL) and batch 4 (mono-culture) were evaluated using two packaging materials (P and NP). Figure 5 shows growth over 21 days at dynamic temperature. No significant differences (*p* > 0.05) were observed between packaging types.

## 4. Discussion

The remarkable inhibition zones (~40 mm) observed in the in vitro assay underscore the potent inhibitory activity of LAB strains against *L. monocytogenes*. These preliminary results were better in contrast with previous studies, where inhibition against foodborne pathogens ranged between 5–16 mm [24,25]. Regarding the in vitro assays, the LAB2-LP15 cocktail showed slightly higher efficacy at 8 °C, but without statistical significance, suggesting modest improvements at higher temperatures.

In cold-smoked trout, LC-LL was the most effective culture at both static and dynamic temperatures, significantly reducing *L. monocytogenes* levels. Delving deeper into the antimicrobial effect of this LAB cocktail, *Lc. carnosum*, one of the strains included, has been described as a producer of leucocin [27]. Bacteriocin production is widely recognised as one of the main mechanisms for inhibiting microbial growth, and in this context, the production of leucocin appears to be the primary factor responsible for the observed inhibitory effect against *L. monocytogenes*, due to its sensitivity to leucocin that has been previously reported by other authors [28,29]. LAB2-LP15 also showed inhibition at 5 °C and, being a known probiotic, offers additional value. In this case, bacteriocin production by this LAB cocktail had previously been assessed, yielding negative results. Therefore, the inhibition of *L. monocytogenes* appears to be closely related to nutrient competition, suggesting an attenuated Jameson effect. However, it was ineffective at dynamic temperatures, likely due to rapid *L. monocytogenes* growth before LAB reached sufficient biomass. LPG1-LAB 119, despite promising in vitro results, failed in the food matrix under both temperature conditions, highlighting the need to evaluate LAB in real products where matrix effects can reduce efficacy when compared with preliminary screenings.

Even with multiple barriers (salt, smoking, vacuum, cold) in control samples (without LAB), *L. monocytogenes* reached a concentration higher than 8 log CFU/g over 22 days, emphasizing the need for bioprotection. The growth rate of the pathogen in mono-culture is in line with results reported by other authors [21,22]. Hence, a similar growth rate was observed in liquid-smoked rainbow trout in 10 days of packaged storage at 4 °C [30], as well as in other fishery products like salmon paté or surimi at cold storage conditions [21,31]. In our study, the pathogen behaved completely differently when co-cultured with LC-LL, as in the latter case, *L. monocytogenes* decreased below the initial levels, and so the growth model was not applicable.

Dynamic temperatures shortened the lag phase and increased the growth rate and final counts, stressing the importance of strict cold chain control.

LAB2-LP15 preserved sensory quality best, while LC-LL and LPG1-LAB119 caused premature spoilage.

No significant differences (*p* > 0.05) were observed in the level of *L. monocytogenes* within the type of culture, in accordance with the results of other authors [32,33], who found similar levels in a study on hot smoked rainbow trout with regular vacuum packaging and MAP (nitrogen) storage at cold temperatures (3–7 °C). This fact may be attributed to the bacterium’s metabolic flexibility as a facultative aerobe. Hence, it is known that *L. monocytogenes* is highly adapted to the anaerobic conditions of the mammalian intestine [34]. Depending on environmental conditions, *L. monocytogenes* can modify its metabolic pathways. This adaptation likely enables the foodborne pathogen to grow in environments with extremely low oxygen concentrations. Based on our results, it can be concluded that the permeability of packaging material is not relevant to guaranteeing the safety of cold-smoked rainbow trout, with other factors needing to be considered in the design of a safe final product.

## 5. Conclusions

Fish and fishery products like salmon (fresh or smoked) have traditionally benefited from the use of LAB as a bioprotection strategy. However, the inhibitory effect of LAB species with probiotic potential has not been tested in RTE fish products. Our study demonstrates that, of the three LAB cocktails tested, the LC-LL LAB cocktail presented the greatest inhibitory effect against *L. monocytogenes* on cold-smoked rainbow trout. However, it is interesting to highlight that the LAB2-LP15 cocktail, with demonstrated probiotic activity, was able to limit the growth of the pathogen at 5 °C, also being the best candidate to preserve the sensory profile of the rainbow trout. Unfortunately, LAB2-LP15 seemed ineffective under conditions of thermal abuse (dynamic temperature 4–20 °C). More research is needed to identify the temperature range at which the LAB2-LP15 cocktail maintains its inhibitory effects. In addition, research into different combinations of LAB cocktails, such as LAB2-LP15 and LC-LL, at varying concentrations, could lead to an optimized blended cocktail assembling their beneficial properties. As discussed previously, reduced-oxygen film in vacuum-packaged fillets was not a critical factor to reduce *L. monocytogenes* growth, and thus, the design of a safe final product should rely on other barriers different from packaging. The use of bioprotective agents has been demonstrated to be an excellent and viable alternative that should be kept as common practice to produce RTE cold-smoked rainbow trout and, in general, smoked fish. The addition of a combined cocktail of LC-LL and LAB2-LP15 represents a promising strategy to produce high-quality, safe, and healthy food.

## Figures and Tables

**Table 1:** Growth parameters were estimated by the Baranyi and Roberts model adjusted to growth data of *L. monocytogenes* cocktail II in mono- and co-culture with LAB cocktails (LPG1-LAB119; LAB2-LP15) in TSB at 5 °C and 8 °C.

Culture	5 °C				8 °C			
	λ (h)	µ_max_ (1/h)	log N_0_	log N_max_	λ (h)	µ_max_ (1/h)	log N_0_	log N_max_
*L. monocytogenes*	11.5 ± 12.7 ^A^	0.019 ± 0.001 ^A^	1.78 ± 0.16 ^AB^	8.88 ± 0.12 ^A^	9.5 ± 13.5 ^A^	0.033 ± 0.003 ^A^	3.45 ± 0.22 ^A^	9.42 ± 0.09 ^A^
*L. monocytogenes* in co-culture with LPG1-LAB119	78.0 ± 10.7 ^B^	0.018 ± 0.001 ^A^	2.13 ± 0.11 ^B^	8.23 ± 0.12 ^B^	31.7 ± 23.7 ^A^	0.027 ± 0.003 ^A^	2.22 ± 0.39 ^B^	9.44 ± 0.23 ^A^
*L. monocytogenes* in co-culture with LAB2-LP15	33.7 ± 12.5 ^A^	0.017 ± 0.001 ^A^	1.44± 0.13 ^A^	7.80 ± 0.12 ^C^	12.4 ± 11.0 ^A^	0.017 ± 0.008 ^A^	3.63 ± 0.47 ^A^	8.58 ± 0.22 ^B^

Values are expressed as mean ± standard error. For each parameter (column), values with different uppercase letters are significantly different (*p* < 0.05).

**Table 2:** Growth parameters estimated by the Baranyi and Roberts model adjusted to growth data of *L. monocytogenes* cocktail II in mono- and co-culture with LAB2-LP15 LAB cocktail in cold smoked rainbow trout at 5 °C and dynamic temperature (4–20 °C).

Culture	Static 5 °C				Dynamic 4–20 °C			
	λ (h)	µ_max_ (1/h)	log N_0_	log N_max_	λ (h)	µ_max_ (1/h)	log N_0_	log N_max_
*L. monocytogenes*	154.93 ± 30.49 ^A^	0.0097 ± 0.0012 ^A^	4.30 ± 0.16 ^A^	7.18 ± 0.27 ^A^	94.32 ± 26.13	0.0125 ± 0.0012	4.68 ± 0.238	8.37 ± 0.205
*L. monocytogenes* in co-culture with LAB2-LP15	137.56 ± 49.78 ^A^	0.0094 ± 0.0072 ^A^	4.36 ± 0.14 ^A^	5.29 ± 0.12 ^B^	NE *	NE	NE	NE

* NE: Not estimated value. Values are expressed as mean ± standard error. For each parameter (column), values with different uppercase letters are significantly different (*p* < 0.05).

**Table 3:** Sensory parameters evaluated in cold-smoked rainbow trout stored at 5 °C.

Culture	Day	Color	Odor	Texture	Oxidation	Flavor	Salt
No inoculated (control)	0	4.73 ± 0.47 ^Aa^	4.91 ± 0.30 ^Aa^	4.91 ± 0.30 ^Aa^	4.73 ± 0.47 ^Aa^	4.89 ± 0.00 ^Aa^	4.70 ± 0.45 ^Aa^
	5	4.25 ± 0.71 ^Aab^	4.63 ± 0.52 ^Aa^	4.88 ± 0.35 ^Aa^	4.25 ± 0.46 ^Aab^	4.75 ± 0.50 ^Aa^	4.20 ± 0.50 ^Aa^
	10	3.60 ± 0.70 ^Abc^	4.40 ± 0.70 ^Aab^	4.40 ± 0.70 ^Aab^	3.90 ± 0.57 ^Abc^	NE *	NE
	14	3.40 ± 0.70 ^Abc^	4.30 ± 0.48 ^Aabc^	3.90 ± 0.74 ^Ab^	3.90 ± 0.57 ^Abc^	NE	NE
	19	3.25 ± 0.71 ^Ac^	3.75 ± 0.52 ^Abc^	3.88 ± 0.83 ^Ab^	3.75 ± 0.71 ^Abc^	NE	NE
	24	3.00 ± 0.53 ^ABc^	3.63 ± 0.46 ^Ac^	3.63 ± 1.06 ^Ab^	3.38 ± 0.52 ^Ac^	NE	NE
*Leuconostoc carnosum* and *Lactococcus lactis* subsp. *lactis* (LC-LL)	0	4.91 ± 0.30 ^Aa^	4.91 ± 0.30 ^Aa^	5.00 ± 0.00 ^Aa^	4.73 ± 0.47 ^Aa^	4.56 ± 0.44 ^Aa^	4.60 ± 0.55 ^Aa^
	5	4.13 ± 0.83 ^Aab^	4.50 ± 0.76 ^Aa^	4.18 ± 0.35 ^Aab^	4.00 ± 0.00 ^Aab^	5.00 ± 1.50 ^Aa^	4.50 ± 0.50 ^Aa^
	10	3.70 ± 0.95 ^Abc^	4.20 ± 0.63 ^Aa^	3.55 ± 0.52 ^Aab^	4.00 ± 0.47 ^Aab^	NE	NE
	14	2.90 ± 0.88 ^Abc^	3.90 ± 0.99 ^Aab^	3.45 ± 1.03 ^Aab^	3.75 ± 0.70 ^Ab^	NE	NE
	19	2.75 ± 1.20 ^Bbc^	2.75 ± 0.88 ^Abc^	2.73 ± 1.07 ^Ab^	3.60 ± 1.06 ^Ab^	NE	NE
	24	2.00 ± 0.89 ^ABd^	2.67 ± 1.16 ^ABc^	2.55 ± 1.16 ^Ab^	3.75 ± 0.46 ^Ab^	NE	NE
*Lactobacillus pentosus* (LPG1-LAB119)	0	4.64 ± 0.67 ^Aa^	4.91 ± 0.30 ^Aa^	4.82 ± 0.40 ^Aa^	4.55 ± 0.69 ^Aa^	4.89 ± 0.00 ^Aa^	4.60 ± 0.45 ^Aa^
	5	4.13 ± 0.64 ^Aab^	4.38 ± 0.74 ^Aab^	4.00 ± 0.52 ^Aab^	3.88 ± 0.35 ^Aab^	4.50 ± 0.50 ^Aa^	4.33 ± 0.50 ^Aa^
	10	3.60 ± 0.97 ^Aab^	3.90 ± 0.74 ^Aabc^	3.36 ± 0.70 ^Aab^	3.80 ± 0.79 ^Aab^	NE	NE
	14	3.50 ± 0.48 ^Abc^	3.70 ± 0.95 ^Abc^	3.36 ± 0.95 ^Aab^	3.60 ± 0.70 ^Ab^	NE	NE
	19	3.30 ± 0.76 ^Abc^	2.89 ± 0.93 ^Acd^	2.64 ± 1.06 ^Ab^	3.50 ± 0.76 ^Ab^	NE	NE
	24	2.50 ± 0.76 ^Bc^	2.25 ± 0.82 ^Bd^	2.55 ± 1.31 ^Ab^	3.39 ± 0.52 ^Ab^	NE	NE
*Lactobacillus pentosus* and *Lactobacillus plantarum* (LAB2-LP15)	0	4.82 ± 0.40 ^Aa^	4.73 ± 0.47 ^Aa^	4.82 ± 0.40 ^Aa^	4.73 ± 0.47 ^Aa^	4.67 ± 0.45 ^Aa^	4.27 ± 0.55 ^Aa^
	5	4.25 ± 0.46 ^Aab^	4.33 ± 0.87 ^Aab^	4.63 ± 0.52 ^Aa^	4.00 ± 0.58 ^Aab^	4.25 ± 1.00 ^Aa^	4.50 ± 0.00 ^Aa^
	10	3.88 ± 0.53 ^Aab^	4.00 ± 0.81 ^Aab^	4.30 ± 0.48 ^Aab^	3.88 ± 0.79 ^Aab^	NE	NE
	14	3.63 ± 0.71 ^Ab^	3.90 ± 0.74 ^Aab^	4.00 ± 0.47 ^Aab^	3.80 ± 0.63 ^Ab^	NE	NE
	19	3.50 ± 0.92 ^Ab^	3.43 ± 0.79 ^Ab^	4.00 ± 0.76 ^Aab^	3.80 ± 0.64 ^Ab^	NE	NE
	24	3.50 ± 0.99 ^Ab^	3.29 ± 0.76 ^ABb^	3.63 ± 1.06 ^Ab^	3.38 ± 0.52 ^Ab^	NE	NE

* NE: Not estimated value. Values are expressed as mean ± standard error. For each parameter (column), values with different uppercase letters indicate differences between treatments (*p* < 0.05) while lowercase letters show significant differences between batches for same attribute (*p* < 0.05).

## Data Availability

The original contributions presented in the study are included in the article, further inquiries can be directed to the corresponding author.

## Abbreviations

RTE	Ready-to-Eat
LAB	Lactic Acid Bacteria
CFU	Colony Forming Unit
NaCl	Sodium Chloride
IL-8	Interleukin 8
IL-6	Interleukin 6
IL-10	Interleukin 10
MRS	de Man, Rogosa and Sharpe
LC-LL	*Leuconostoc carnosum* and *Lactococcus lactis* subsp. *lactis*
RCF	Relative Centrifugal Force
CSIC	Consejo Superior de Investigaciones Científicas
CECT	Spanish Type Culture Collection
BCCM	Belgian Coordinated Collections of Microorganisms
ANSES	French Agency for Food, Environmental and Occupational Health & Safety
TSB	Tryptone Soya Broth
PA	Polyamide
PE	Polyethylene
EVOH	Ethylene Vinyl Alcohol
MAP	Modified Atmosphere Packaging
QIM	Quality Index Method
µ_max_	Maximum Specific Growth Rate
N_0_	Initial Cell Concentration
N_max_	Maximum Population Density
EU	European Union
EFSA	European Food Safety Authority
CTAQUA	Andalusian Aquaculture Technological Centre
CO_2_	Carbon Dioxide
